# Molecular and cellular identification of the immune response in peripheral ganglia following nerve injury

**DOI:** 10.1186/s12974-018-1222-5

**Published:** 2018-06-26

**Authors:** Jane A. Lindborg, Jon P. Niemi, Madeline A. Howarth, Kevin W. Liu, Christian Z. Moore, Deepti Mahajan, Richard E. Zigmond

**Affiliations:** 1 Department of Neurosciences, School of Medicine, Case Western Reserve University, Cleveland, OH USA; 2 Science and Engineering Program, Hathaway Brown School, Shaker Heights, OH USA; 3 Present Address: Department Neurosciences, School of Medicine, 10900 Euclid Avenue, Robbins E701, Cleveland, OH 44106-4975 USA

**Keywords:** Axotomy, Dorsal root ganglion (DRG), Macrophage, Neuroinflammation, Neutrophil, Regeneration, Superior cervical ganglion (SCG)

## Abstract

**Background:**

Neuroinflammation accompanies neural trauma and most neurological diseases. Axotomy in the peripheral nervous system (PNS) leads to dramatic changes in the injured neuron: the cell body expresses a distinct set of genes known as regeneration-associated genes, the distal axonal segment degenerates and its debris is cleared, and the axons in the proximal segment form growth cones and extend neurites. These processes are orchestrated in part by immune and other non-neuronal cells. Macrophages in ganglia play an integral role in supporting regeneration. Here, we explore further the molecular and cellular components of the injury-induced immune response within peripheral ganglia.

**Methods:**

Adult male wild-type (WT) and *Ccr2*^*−/−*^ mice were subjected to a unilateral transection of the sciatic nerve and axotomy of the superior cervical ganglion (SCG). Antibody arrays were used to determine the expression of chemokines and cytokines in the dorsal root ganglion (DRG) and SCG. Flow cytometry and immunohistochemistry were utilized to identify the cellular composition of the injury-induced immune response within ganglia.

**Results:**

Chemokine expression in the ganglia differed 48 h after nerve injury with a large increase in macrophage inflammatory protein-1γ in the SCG but not in the DRG, while C-C class chemokine ligand 2 was highly expressed in both ganglia. Differences between WT and *Ccr2*^*−/−*^ mice were also observed with increased C-C class chemokine ligand 6/C10 expression in the WT DRG compared to C-C class chemokine receptor 2 (CCR2)^−/−^ DRG and increased CXCL5 expression in CCR2^−/−^ SCG compared to WT. Diminished macrophage accumulation in the DRG and SCG of *Ccr2*^*−/−*^ mice was found compared to WT ganglia 7 days after nerve injury. Interestingly, neutrophils were found in the SCG but not in the DRG. Cytokine expression, measured 7 days after injury, differed between ganglion type and genotype. Macrophage activation was assayed by colabeling ganglia with the anti-inflammatory marker CD206 and the macrophage marker CD68, and an almost complete colocalization of the two markers was found in both ganglia.

**Conclusions:**

This study demonstrates both molecular and cellular differences in the nerve injury-induced immune response between DRG and SCG and between WT and *Ccr2*^*−/−*^ mice.

## Background

While the inflammatory response to injury in the peripheral nervous system (PNS) is complex and involves a number of immune cells, including dendritic, T, and B cells [1, 2], it is largely dominated by macrophages [3]. Macrophage responses to a peripheral axonal injury occur in two distinct compartments: the nerve distal to the injury site and the axotomized ganglion. The largest contribution to macrophage accumulation within injured PNS tissues come from monocyte-derived macrophages. In the sciatic nerve 7 days after injury, infiltrating macrophages outnumber the resident population 3:1 [4]. Infiltration of the sciatic nerve begins 48 h after injury with peak accumulation occurring between 7 and 14 days [3, 5]. Additionally, macrophage numbers remain elevated in the nerve for at least 30 days after injury [6].

In peripheral ganglia, monocyte infiltration and macrophage accumulation follow a similar timeline to that seen in the sciatic nerve [7–9]. In the superior cervical ganglion (SCG), monocyte-derived macrophages are present at 48 h after injury and remain for at least 2 weeks following transection of the internal and external carotid nerves [9, 10]. Likewise, the lumbar dorsal root ganglion (DRG) shows accumulation beginning 48 to 72 h after sciatic nerve injury with an increased number of monocyte-derived macrophages remaining for at least 28 days [11, 12].

The signal-mediating monocyte/macrophage entry into PNS tissue following injury is thought to be the C-C motif chemokine ligand 2 (CCL2), as a global knockout of its primary receptor C-C class chemokine receptor 2 (CCR2) yields a significant decrease in macrophage accumulation in the sciatic nerve following injury [13, 14]. CCL2, also known as monocyte chemoattractant protein-1 (MCP-1), recruits monocytes to sites of inflammation, infection, and injury. CCL2 also has the ability to act as a chemokine for neutrophils, T cells, and dendritic cells [15, 16].

While the function of macrophages in peripheral ganglia following nerve injury has been unknown until recently, evidence in the DRG and SCG indicates a role in axonal regeneration [11, 14, 17, 18]. Since there is very little cell death and degeneration occurring in peripheral ganglia after injury [19, 20], the stereotypical phagocytic function of macrophages is likely not their main function in the cell body compartment after axonal injury, suggesting an alternate purpose [21, 22]. Recent work by our laboratory has demonstrated that macrophage accumulation near injured neuronal cell bodies is both necessary and sufficient for peripheral axon regeneration [14, 17]. By inhibiting macrophage accumulation within the SCG and DRG following axotomy using a global knockout of the chemokine receptor *Ccr2* (*Ccr2*^*−/−*^), we showed that in vitro axonal regeneration is significantly reduced when the injury-induced immune response in ganglia is impaired [14].

Numerous studies on peripheral nerve regeneration and the neuronal cell body response to axonal injury exclusively utilize injury to the sciatic nerve and DRG as a model system. However, the DRG is a very unique ganglion in that the sensory neurons lack dendrites and synaptic input within the cell body region, the neurons within the DRG are a heterogeneous population composed of multiple subtypes, and the ganglia lack a blood-ganglion barrier [23]. The differences between the DRG and other peripheral ganglia could allow for unique mechanisms to arise in response to peripheral axonal injury. Thus, a comparison of the injury responses, and more specifically the axotomy-induced immune responses, between the DRG and other peripheral ganglia could identify cellular and molecular mechanisms that are common elements of peripheral injury or unique elements that are specific to the DRG. The SCG, the largest sympathetic ganglia, has many characteristics that directly oppose those of the DRG such as the presence of presynaptic innervation within the ganglia, a more homogeneous neuronal population, and the existence of a blood-ganglion barrier [24–26].

Nevertheless, very little is known about the complete molecular and cellular components of the injury-induced immune response within peripheral ganglia. Here, we utilize unbiased antibody protein arrays, flow cytometry, and immunohistochemistry (IHC) to identify and characterize the molecular and cellular profile of the immune response after nerve injury within the DRG and SCG of wild-type (WT) and *Ccr2*^*−/−*^ mice.

## Methods

### Mice

Male (8–12-week-old) WT (C57BL/6J; The Jackson Laboratory, Bar Harbor, ME, USA) and *Ccr2*^*−/−*^ (B6.129S4-*Ccr2*^*tm1Ifc*^/J backcrossed to C57BL/6J; The Jackson Laboratory) mice were used for this study. All mice had ad libitum access to food and water and were housed under a 12-h light/dark cycle.

### Injury model

Mice were anesthetized under isoflurane, and the external carotid nerve (ECN) and internal carotid nerve (ICN) of the right SCG were exposed and transected. For axotomy of neurons in the fourth, fifth, and sixth DRG, the right sciatic nerve was exposed and transected at the hip level, and 1 mm of the nerve was removed. The nerves on the left side of the animal were exposed but not transected, and the ganglia served as sham-operated controls. One, two, three, or seven days after injury, mice were killed by CO_2_ inhalation, and the SCG and a combination of the L4, L5, and L6 DRG were harvested for analysis. All surgical procedures were approved by the Case Western Reserve University Institutional Animal Care and Use Committee.

### Antibody arrays

L4 and L5 DRG and SCG from WT and *Ccr2*^*−/−*^ mice were collected at 48 h (for chemokine assay) and 7 days (for cytokine assay) after right sciatic nerve transection and right SCG axotomy and flash frozen for chemokine and cytokine determination by Proteome Profiler Array ARY020 and ARY006 (R&D Systems, Minneapolis, MN, USA), respectively. Ganglia from five mice/group were pooled for each sample, and protein was isolated through homogenization in RIPA buffer (Santa Cruz Biotechnology, Dallas, TX, USA). Protein (180 μg per sample) was diluted and mixed with a cocktail of biotinylated detection antibodies and then incubated with mouse chemokine or cytokine array membrane. After washing, streptavidin-horseradish peroxidase and chemiluminescent detection reagents were added. Array images were obtained using a LI-COR Odyssey Fc Imaging System (LI-COR Biosciences, Lincoln, NE, USA) with a 10-min exposure time and analyzed by densitometry for integral optical density using ImageJ software. The optical density of each pair of chemokine or cytokine spots was normalized to the corresponding positive control spots. The data were then represented as a fold increase of the injured chemokine or cytokine expression compared to the WT sham results. Three arrays were run per condition, and the results were averaged. *t* tests were used to evaluate statistical differences between injured and uninjured tissues within a genotype and in the injured tissue between genotypes. The following chemokines were assessed at 48 h post injury in the DRG and SCG: 6Ckine, BLC, C10, C5/C5a, CCL28, chemerin, CTACK, CXCL16, eotaxin, CX_3_CL1, interleukin (IL)-16, IP-10, I-TAC, CCL2, CXCL1, LIX, CCL8/MCP-2, MCP-5, MDC, MIG, macrophage inflammatory protein-1 (MIP-1)α/β, macrophage inflammatory protein-1 gamma (MIP-1γ), MIP-2, RANTES, and SDF-1. The following cytokines were assessed at 7 days post injury in the DRG and SCG: CXCL13, C5a, G-CSF, GM-CSF, CCL1, CCL11, soluble intercellular adhesion molecule-1 (sICAM-1), IFN-γ, IL-1α, IL-1β, interleukin-1 receptor antagonist (IL-1ra), IL-2, IL-3, IL-4, IL-5, IL-6, IL-7, IL-10, IL-13, IL-12p70, IL-16, IL-17, IL-23, IL-27, IP-10, CXCL11, KC, M-CSF, CCL2, CCL12, CXCL9, CCL3, CCL4, CXCL2, CCL5, CXCL12, CCL17, tissue inhibitor of metalloproteinase-1 (TIMP-1), tumor necrosis factor alpha (TNF-α), and TREM-1.

### Flow cytometry

A pooled sample of L4, L5, and L6 DRG and single SCG was enzymatically digested in 0.125% collagenase for 1 h at 37 °C. Mechanical digestion using a 23-gauge needle attached to a 1-ml syringe produced single cell suspensions which were filtered through a 35-μm cell strainer. Dead cells were labeled using a Live/Dead Fixable Blue Dead Cell Stain kit (Thermo Fisher Scientific, Waltham, MA, USA) for 30 min at 4 °C. Cells were then washed in FACS buffer (PBS, 1% BSA) and blocked with a monoclonal antibody to CD16/CD32 (1:500; Thermo Fisher Scientific) for 10 min at 4 °C. Cells were incubated with fluorophore-conjugated antibodies against F4/80, CD11b, Ly6G (1A8), and p75 ([F4/80] Cat no. 123130; [CD11b] Cat no. 101206; [Ly6G] Cat no. 127610; [p75] Cat no. 113405) for 1 h at 4 °C. Cells were washed and resuspended in FACS buffer and then run on a BD FACSAria (BD Biosciences, San Jose, CA, USA) and analyzed using FlowJo (BD Biosciences). CD45 was not used to gate leukocyte populations, and therefore, non-leukocyte populations may be included in the analysis [27]. All events were gated based on viable single cells. Compensation and gating were performed using negative, single-stained, and isotype controls. Cell populations were gated as follows: F4/80^+^CD11b^+^ (macrophages), CD11b^+^Ly6G^−^ (monocytes/macrophages), CD11b^+^Ly6G^+^ (neutrophils), and p75^+^ (Schwann cells).

### IHC

L5 DRG and SCG from WT and *Ccr2*^*−/−*^ mice were removed 1, 3, or 7 days post injury, and the ganglia were desheathed and fixed by immersion in 4% paraformaldehyde. The tissues were cryoprotected in 30% sucrose and embedded in Tissue-Tek O.C.T. compound (Electron Microscopy Sciences, Hatfield, PA, USA). IHC was performed on 10-μm cryostat sections to double label tissue with a macrophage marker, CD68, and an anti-inflammatory macrophage marker, CD206. A goat polyclonal antibody to CD206 (1:200; R&D Systems) was incubated with tissue sections overnight at 4 °C. After washing, the sections were incubated in Alexa Fluor 488 secondary antibody (1:400; Thermo Fisher Scientific) for 1 h and washed in PBS. Next, a rat monoclonal antibody against CD68 (1:200; AbD Serotec, Oxford, UK) was incubated with tissue sections overnight at 4 °C. After additional washes, the sections were incubated with a Cy3 secondary antibody (1:200; Jackson ImmunoResearch Laboratories, Inc., West Grove, PA, USA) for 1 h. The sections were then stained with DAPI (1:1000; Thermo Fisher Scientific). IHC was also performed to label neutrophils in the ganglia using a rabbit polyclonal antibody to myeloperoxidase (MPO; 1:100; Abcam, Cambridge, UK) overnight at 4 °C and a Cy3 donkey anti-rabbit secondary antibody (1:400; Jackson ImmunoResearch Laboratories, Inc.). Additionally, IHC was performed on L3, L4, L5, and L6 DRG and SCG to identify the percentage of injured neurons after axotomy using a mouse monoclonal anti-HuC/D (1:100; Thermo Fisher Scientific) and a rabbit polyclonal anti-activating transcription factor 3 (ATF3) antibody (1:100; Sigma-Aldrich, St. Louis, MO, USA) by incubating sections overnight at 4 °C. Prior to the addition of primary ATF3 and HuC/D antibodies, the tissue underwent citrate buffer antigen retrieval and an endogenous mouse antibody blocking step by incubating the tissue with a donkey anti-mouse-unconjugated IgG (1:400; Jackson ImmunoResearch Laboratories, Inc., West Grove, PA, USA). ATF3 and HuC/D were visualized by incubation with goat anti-rabbit Alexa Fluor 594 (1:400; Jackson ImmunoResearch Laboratories, Inc.) and goat anti-mouse IgG2a Alexa Fluor 488 (1:400; Thermo Fisher Scientific). In all experiments, sections not exposed to the primary antibody were included for each experimental group. Images were captured at × 10 or × 25 magnification using HCImage software (Hamamatsu Corporation, Bridgewater, NJ, USA), and then cell counts were performed using ImageJ software. Cell counts were performed to quantify the number of CD68^+^, CD206^+^, CD68^+^CD206^+^, HuC/D^+^, ATF3^+^, and HuC/D^+^ATF3^+^ cells. Data are represented as the sum of the cells counted from three distinct images per ganglion per animal. Five samples were included for each group.

### Statistical analysis

Data in graphs are presented as mean ± SEM. For experiments involving multiple genotypes, treatments, and time points, a two-way ANOVA was performed, followed by a Tukey’s post hoc test. For independent two-group experiments, an unpaired Student’s *t* test (two-tailed) was used to determine statistical significance. Statistical analyses were performed using SigmaPlot, version 12.3 (Systat Software, Inc., San Jose, CA, USA). Values were considered statistically significant at *p* < 0.05. The number of experimental replicates (*n*), the designation of *n* belonging to the number of animals, and experiment repetition are indicated in the figure legends. Data from all experiments were included in the analysis. IHC experiments were performed with the experimenter blinded to genotype.

## Results

### SCG axotomy and sciatic nerve transection injury paradigms

**Fig. 1:**
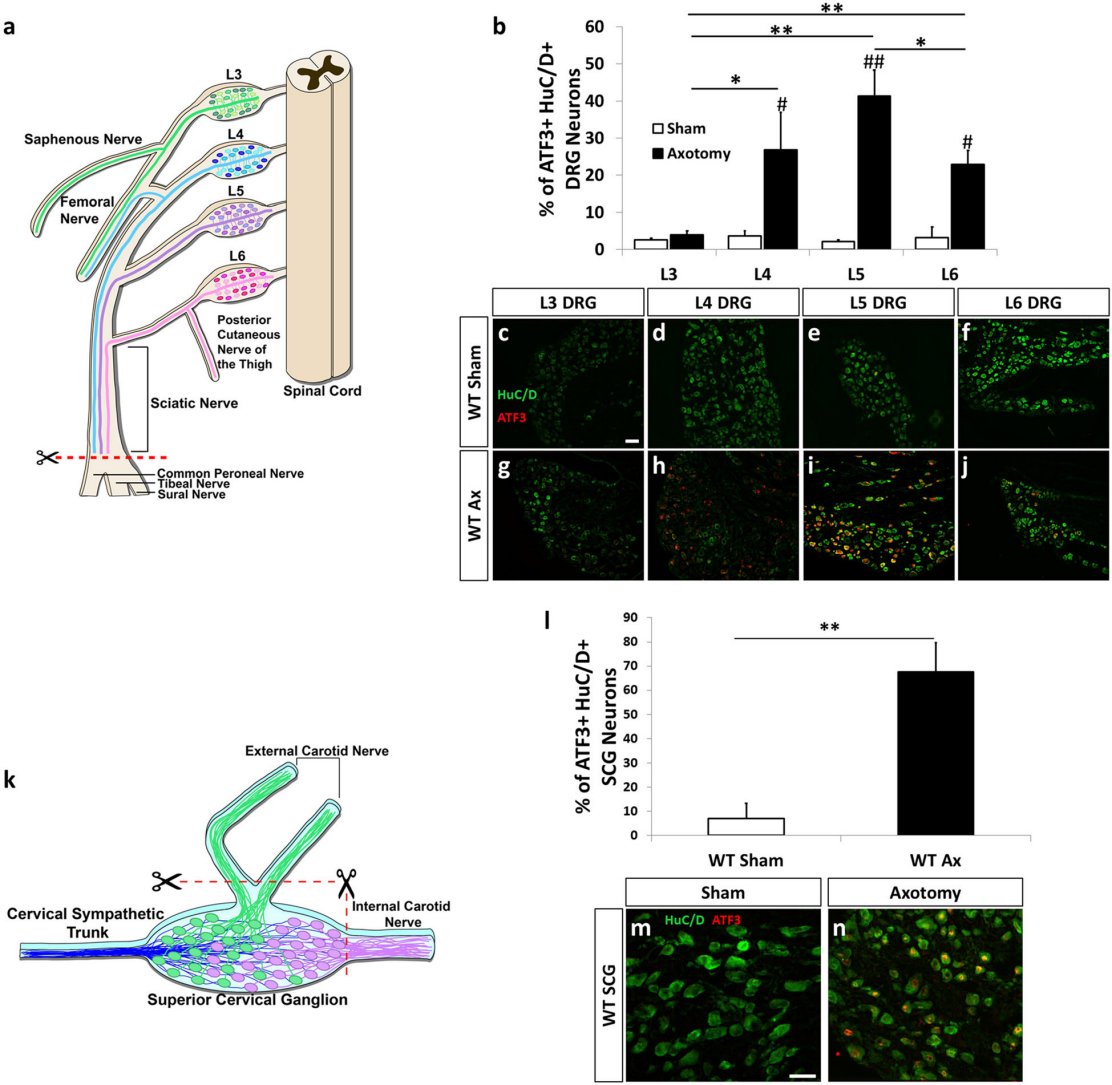
The axonal injury-induced marker ATF3 is highly upregulated in L4, L5, and L6 DRG and in the SCG 7 days after axotomy. A diagram illustrating the sciatic nerve transection injury and the relative innervation of the sciatic nerve by the individual lumbar DRG (**a**). WT mice underwent a unilateral transection of the right sciatic nerve and lumbar DRG were removed and stained for the injury marker ATF3 and the neuronal marker HuC/D. The percentage of ATF3^+^HuC/D^+^ neurons were quantified 7 days after injury (**b**). Representative images of sham-operated (**c**–**f**) and axotomized (**g**–**j**) DRG for L3 (**c**, **g**), L4 (**d**, **h**), L5 (**e**, **i**), and L6 (**f**, **j**) DRG are shown. A diagram illustrating the SCG axotomy and the relative innervation of the external and internal carotid nerves (**k**). In the SCG diagram, blue represents the preganglionic fibers, the green cell bodies are neurons that project into the ECN, and the purple cell bodies project into the ICN. Mice underwent a unilateral transection of the ECN and ICN of the right SCG. SCG were removed 7 days post injury and stained with ATF3 and HuC/D. The percentage of ATF3^+^ HuC/D^+^ SCG neurons were quantified (**l**). Representative images of sham-operated (**m**) and axotomized (**n**) SCG are shown. The data are presented as the mean ± SEM. DRG images were taken at × 10 magnification. SCG images were taken at × 25 magnification. Scale bar = 50 μm. *n* = 5 per group. *p < 0.05. **p < 0.001 between groups. ^#^*p* < 0.05. ^##^*p* < 0.001 between sham and axotomy within groups

Complete or partial injury to the sciatic nerve is one of the most widely used models to study peripheral nerve regeneration, degeneration, and neuropathic pain [28]. The sciatic nerve is a mixed nerve containing sensory, motor, and sympathetic axons. Sensory axons from the DRG at lumbar levels L4, L5, and L6 project into the sciatic nerve [29, 30]. To examine the injury-induced neuroimmune response within the lumbar DRG, we employed a sciatic nerve transection to injure neuronal cell bodies of L4, L5, and L6 DRG (Fig. 1a). ATF3 is a transcription factor that is highly upregulated in injured DRG and SCG neurons [31, 32]. To determine the extent of injury in the DRG at different lumbar levels, L3, L4, L5, and L6 DRG were collected 7 days after a sciatic nerve transection or sham operation and colabeled with ATF3 and the neuronal marker HuC/D [33]. L4, L5, and L6 DRG all showed significant increases in ATF3 with 26.9, 41.3, and 22.1% of HuC/D^+^ neurons expressing the injury marker, respectively (Fig. 1b–j). The L3 DRG did not exhibit ATF3 upregulation after sciatic nerve transection (Fig. 1b, c, g).

To study the injury-induced cellular and molecular neuroimmune changes in the SCG, the ECN and ICN, the two major postganglionic nerves of the ganglia, were transected (Fig. 1k). A previous study utilizing retrogradely transported horseradish peroxidase to identify the localization of SCG neurons that project into the ICN and ECN found that when both nerves were cut, approximately 80% of the SCG neurons were injured [34]. SCG were harvested 7 days after axotomy or a sham surgery and colabeled with HuC/D and ATF3. In the SCG, 67.5% of HuC/D^+^ sympathetic neurons expressed ATF3 7 days after injury (Fig. 1l–n).

### Chemokine expression in the axotomized ganglia

**Fig. 2:**
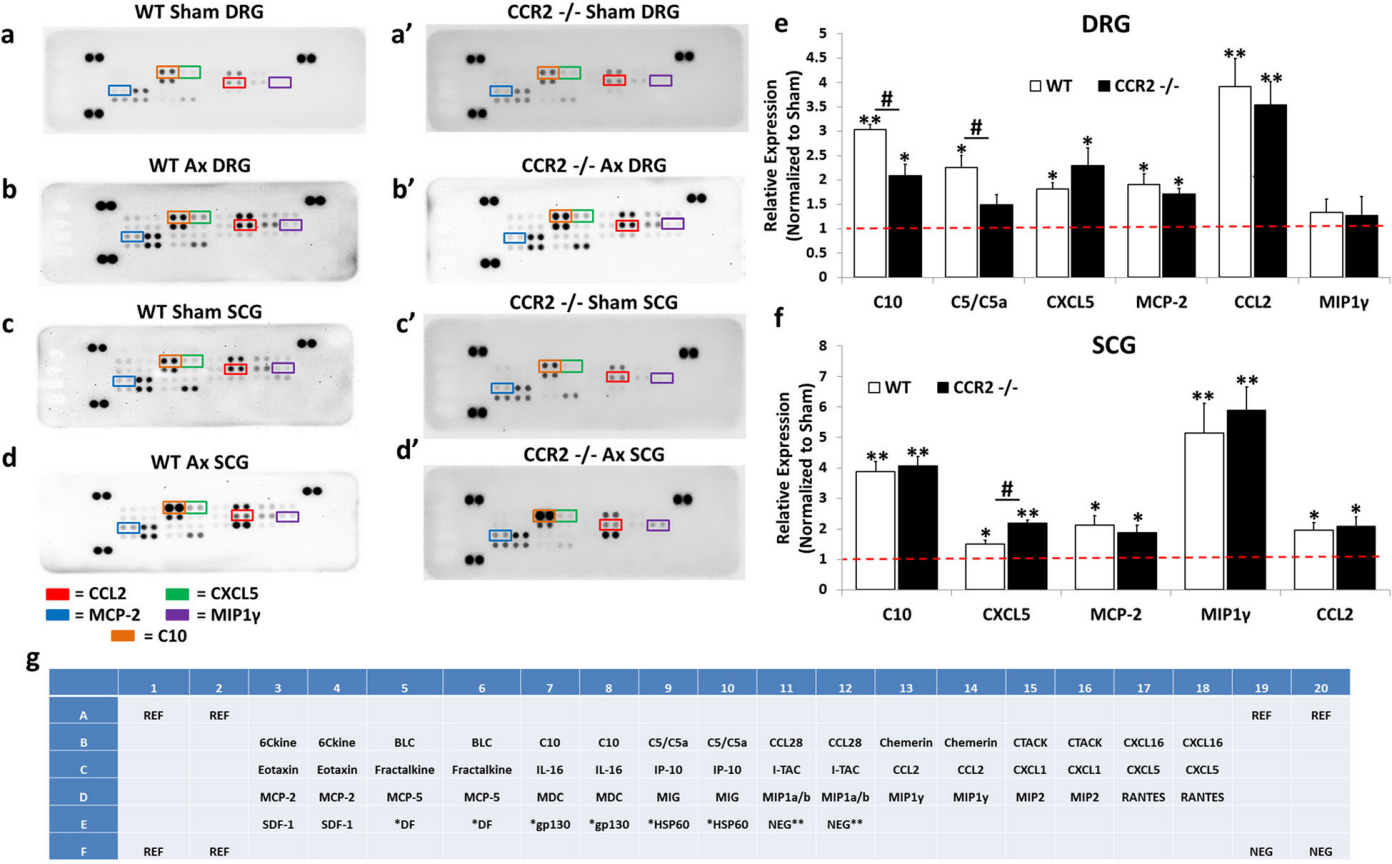
Chemokines are differentially expressed in the DRG and SCG 48 h after axotomy. Mice underwent a unilateral transection of the right sciatic nerve and the right ICN and ECN of the SCG. Forty-eight hours after axotomy, the injured and contralateral sham-operated L4/L5 DRG and SCG were collected and flash frozen. Protein was isolated from sham and injured ganglia pooled from five mice and used for measurement of chemokines using a Proteome Profiler Mouse Array kit (**g**). Representative arrays for WT [85] and *Ccr2*^*−/−*^ (**a’**–**d’**) mice for DRG (**a**, **a’**, **b**, **b’**) and SCG (**c**, **c’**, **d**, **d’**) are shown. Array images were analyzed by densitometry for integral optical density using ImageJ. The optical density for each cytokine was then normalized to the internal control for each blot. The data are represented as the fold increase in the expression of the injured condition compared to the sham for each genotype for DRG (**e**) and SCG (**f**). The spots corresponding to the cytokines CCL2, CXCL5, CCL8/MCP-2, MIP-1γ, and CCL6/C10 are highlighted in the representative arrays based on the template indicating the location of controls and various chemokine capture antibodies (**g**). *N* = 3 arrays per group. The data are presented as mean ± SEM. *p < 0.05. **p < 0.001 represents significance of expression relative to sham within genotype. ^#^*p* < 0.05. ^##^*p* < 0.001 represents significance between genotypes

To begin to assess the inflammatory response that occurs near injured neuronal cell bodies following peripheral nerve injury, we first measured the expression of chemokines, the proteins which play a critical role in immune cell recruitment to tissues [35]. Macrophages, the major immune cell type studied in peripheral nerve injury, have been shown to accumulate in the DRG and SCG within 48 to 72 h after nerve injury [9, 12]. Therefore, we assessed the expression of chemokines in ganglia using antibody arrays in WT and *Ccr2*^*−/−*^ mice 48 h after axotomy. In the DRG, WT mice displayed significant injury-induced expression of multiple chemokines and immune-related factors including a 3.0-fold increase in CCL6/C10, a 2.2-fold increase in complement cascade component C5/C5a, a 1.8-fold increase in CXCL5, a 1.9-fold increase in CCL8/MCP-2, and a 3.9-fold increase in CCL2 compared to WT sham DRG (Fig. 2a, b, e). Axotomized *Ccr2*^*−/−*^ mice exhibited a significant 2.1-fold increase in CCL6/C10, a 2.3-fold increase in CXCL5, a 1.7-fold increase in CCL8/MCP-2, and a 3.5-fold increase in CCL2 compared to *Ccr2*^*−/−*^ sham DRG (Fig. 2a’ , b’ , e). Comparison of the DRG chemokine expression profiles between genotypes found that CCL6/C10 and C5/C5a were more highly expressed in the axotomized WT DRG compared to *Ccr2*^*−/−*^ DRG (Fig. 2e).

In the SCG, WT mice displayed significant injury-induced expression of multiple chemokines and immune-related factors including a 3.9-fold increase in CCL6/C10, a 1.5-fold increase in CXCL5, a 2.1-fold increase in CCL8/MCP-2, a 5.1-fold increase in MIP-1γ, and a 1.9-fold increase in CCL2 compared to WT sham SCG (Fig. 2c, d, f). Axotomized *Ccr2*^*−/−*^ mice exhibited a significant 4.1-fold increase in CCL6/C10, a 2.2-fold increase in CXCL5, a 1.9-fold increase in CCL8/MCP-2, a 5.9-fold increase in MIP-1γ, and a 2.1-fold increase in CCL2 compared to *Ccr2*^*−/−*^ sham DRG (Fig. 2c’ , d’ , f). A comparison of the SCG injury-induced chemokine expression profiles between genotypes found that CXCL5 was more highly expressed in the *Ccr2*^*−/−*^ SCG compared to the WT SCG 48 h after injury (Fig. 2f). The most prominent differences in expression patterns between DRG and SCG are in C5/C5a and MIP-1γ. C5/C5a is significantly increased in the WT DRG but is not upregulated in WT or Ccr2^−/−^ SCG 48 h after injury (Fig. 2e, f). Alternately, MIP-1γ shows a greater than fivefold upregulation in the injured WT and *Ccr2*^*−/−*^ SCG but is not increased in the DRG after injury (Fig. 2e, f).

### Cellular analysis of non-neuronal populations in the injured ganglia

**Fig. 3:**
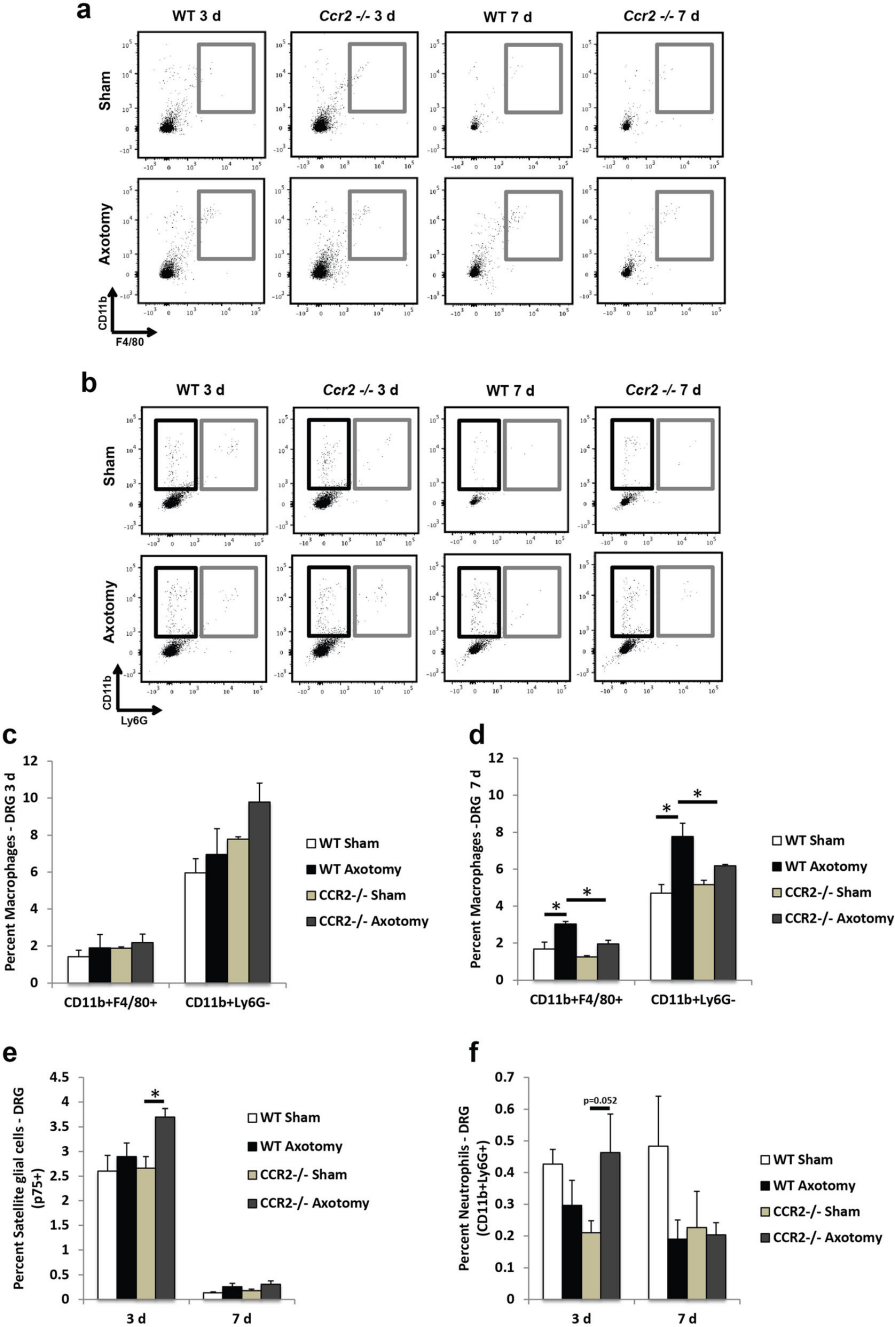
Macrophage accumulation is significantly diminished in the axotomized DRG of *Ccr2*^*−/−*^ mice compared to WT mice, while satellite glial cells and neutrophils are comparable between genotypes. Three and seven days after sciatic nerve transection, L4, L5 and L6 DRG were dissected and examined using flow cytometry. Both sham and axotomized nerves were analyzed. Gray boxes in **a** indicate CD11b^+^F4/80^+^ cells. In **b**, black boxes indicate CD11b^+^Ly6G^−^ cells and gray boxes indicate CD11b^+^Ly6G^+^ cells. Flow cytometric analysis of macrophage populations in the injured DRG (**a**, **b**) shows similar percentages of CD11b^+^F4/80^+^ and CD11b^+^Ly6G^−^ cells between genotypes at 3 days (**c**) and significant attenuation in *Ccr2*^*−/−*^ mice compared to WT mice at 7 days (**d**) post injury. A significant increase in the percentage of satellite glial cells after axotomy was seen in 3 days in *Ccr2*^*−/−*^ DRG alone, with a trend towards a significant increase over injured WT DRG (**e**). No differences were observed for CD11b^+^Ly6G^+^ neutrophils after injury in WT mice, while a trend towards a significant increase was observed 3 days after injury in *Ccr2*^*−/−*^ mice (**f**). Mean ± SEM, two-way ANOVA, Tukey’s post hoc test corrected (within individual time points). *p < 0.05. **p < 0.001. *n* = 3 mice per genotype per time point

Since analysis of chemokine protein expression between axotomized WT and *Ccr2*^*−/−*^ ganglia revealed differences in the relative expression of macrophage and neutrophil chemoattractants, we used flow cytometry to determine whether these differences translate into differential cellular accumulations within the ganglia after injury. We observed an increase in CCL2 expression 2 days after injury in both WT and *Ccr2*^*−/−*^ DRG (Fig. 2e); however, significant increases in macrophage accumulation (CD11b^+^F4/80^+^ and CD11b^+^Ly6G^−^) were not yet seen for either genotype 3 days after injury (Fig. 3a–c). At 7 days after injury and in line with previous findings that used immunohistochemical analysis to identify macrophage populations in the axotomized DRG [14], flow cytometry confirmed a robust increase in macrophages in the injured WT DRG over its sham-operated control (80.0 ± 1.7% for CD11b^+^F4/80^+^ macrophages). While macrophage accumulation was also significantly enhanced in the axotomized *Ccr2*^*−/−*^ DRG over its sham-operated control (55.9 ± 1.9%), the injured WT DRG displayed a more robust increase in CD11b^+^F4/80^+^ macrophages compared to injured *Ccr2*^*−/−*^ DRG (55.5 ± 0.4%, Fig. 3d).

Neutrophil accumulation (CD11b^+^Ly6G^+^) in the DRG was negligible for both genotypes at either time point (Fig. 3b, f). In addition to macrophages, satellite glial cells are another population of non-neuronal cells that are abundant in sensory and sympathetic ganglia [36–38]. Satellite glial cells localize to and surround neurons, and it has been suggested that these cells regulate neuronal function by releasing trophic factors, bradykinin, and other proteins [38–40]. p75 is a neurotrophin receptor that is downregulated by neurons after injury and conversely upregulated by satellite glial cells that surround the neurons [37, 41]. Increased p75 expression in satellite glial cells has been observed in both the ipsilateral and contralateral ganglia after injury [42]. Here, the percent of p75^+^ satellite glial cells in the DRG were comparable between genotypes at both time points, and axotomy induced an increase in p75^+^ cells after 3 days in *Ccr2*^*−/−*^ DRG alone (Fig. 3e). p75^+^ cells were absent in all DRG 7 days after injury, indicating reduced expression in neuronal and glial populations.

**Fig. 4:**
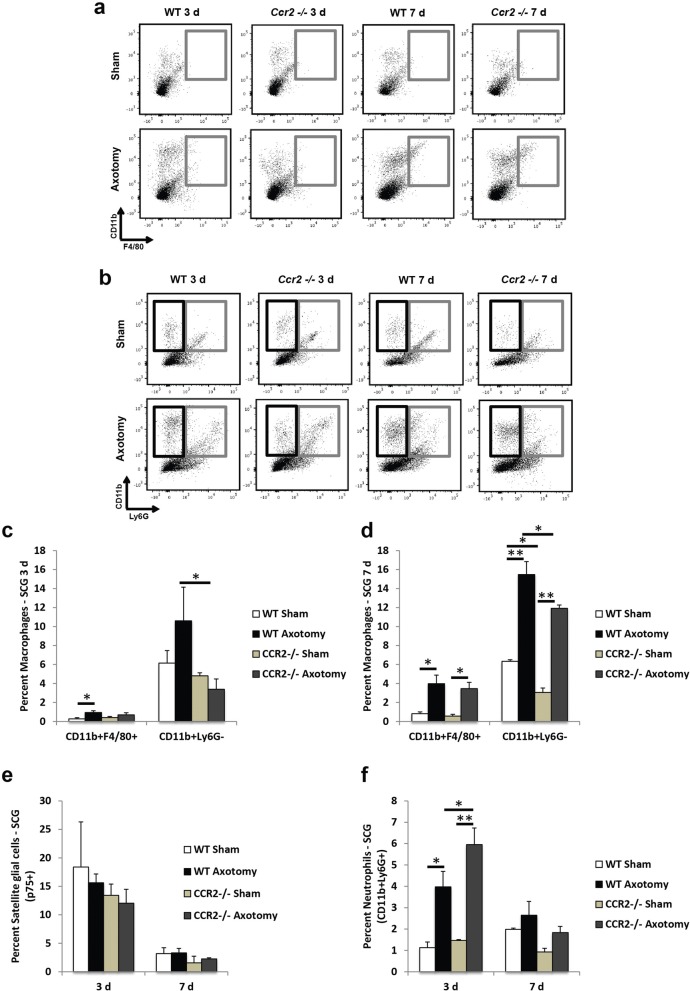
Macrophage accumulation is attenuated in the axotomized SCG of *Ccr2*^*−/−*^ mice compared to WT mice, while *Ccr2*^*−/−*^ SCG show a larger increase in neutrophil accumulation 3 days after injury, compared to the WT SCG. Three and seven days after axotomy, SCG were dissected and examined using flow cytometry. Both sham and axotomized nerves were analyzed. Gray boxes in **a** indicate CD11b^+^F4/80^+^ cells. In **b**, black boxes indicate CD11b^+^Ly6G^−^ cells and gray boxes indicate CD11b^+^Ly6G^+^ cells. Flow cytometric analysis of macrophage populations in the injured SCG (**a**, **b**) indicates increased percentages of CD11b^+^F4/80^+^ cells 3 days after axotomy in WT mice alone and a significant increase in CD11b^+^Ly6G^−^ cells in injured WT mice compared to *Ccr2*^*−/−*^ mice (**c**). Axotomy induced significant increases in macrophages in both genotypes 7 days post injury, with WT mice displaying a larger increase in CD11b^+^Ly6G^−^ cells over *Ccr2*^*−/−*^ mice (**d**). No changes were observed between genotypes in the satellite glial cell population at either time point (**e**). Significant increases in CD11b^+^Ly6G^+^ neutrophils were seen in WT and *Ccr2*^*−/−*^ SCG 3 days post injury (**f**). Neutrophils were more prevalent in the *Ccr2*^*−/−*^ SCG 3 days after axotomy compared to the WT SCG. Mean ± SEM, two-way ANOVA, Tukey’s post hoc test corrected (within individual time points). *p < 0.05. **p < 0.001. *n* = 3 mice per genotype per time point

Evaluating the macrophage response in the injured SCG indicated an increase in CD11b^+^F4/80^+^ cells in the axotomized WT ganglia compared to sham 3 days post injury, and a larger increase in CD11b^+^Ly6G^−^ cells in the injured WT SCG relative to injured *Ccr2*^*−/−*^ SCG (Fig. 4a–c). Similar to prior studies using CD11b^+^ immunostaining [14], flow cytometry revealed a macrophage response to injury in the axotomized *Ccr2*^*−/−*^ SCG compared to its sham control 7 days post injury (Fig. 4d). But while CD11b^+^Ly6G^−^ and CD11b^+^F4/80^+^ macrophage accumulation was significantly increased 7 days after injury in the *Ccr2*^*−/−*^ SCG, the axotomized WT SCG displayed a substantial increase in CD11b^+^Ly6G^−^ macrophages compared to the mutant (15.5 ± 1.4% for WT SCG and 11.9 ± 0.3% for *Ccr2*^*−/−*^ SCG, Fig. 4d). Furthermore, uninjured WT SCG boast a significantly higher population of CD11b^+^Ly6G^−^ resident macrophages compared to uninjured *Ccr2*^*−/−*^ SCG (6.3 ± 0.2% for WT SCG and 3.0 ± 0.5% for *Ccr2*^*−/−*^ SCG, Fig. 4d). Injury produced no change in the expression of p75^+^ satellite glial cells for either genotype at either time point (Fig. 4e).

**Fig. 5:**
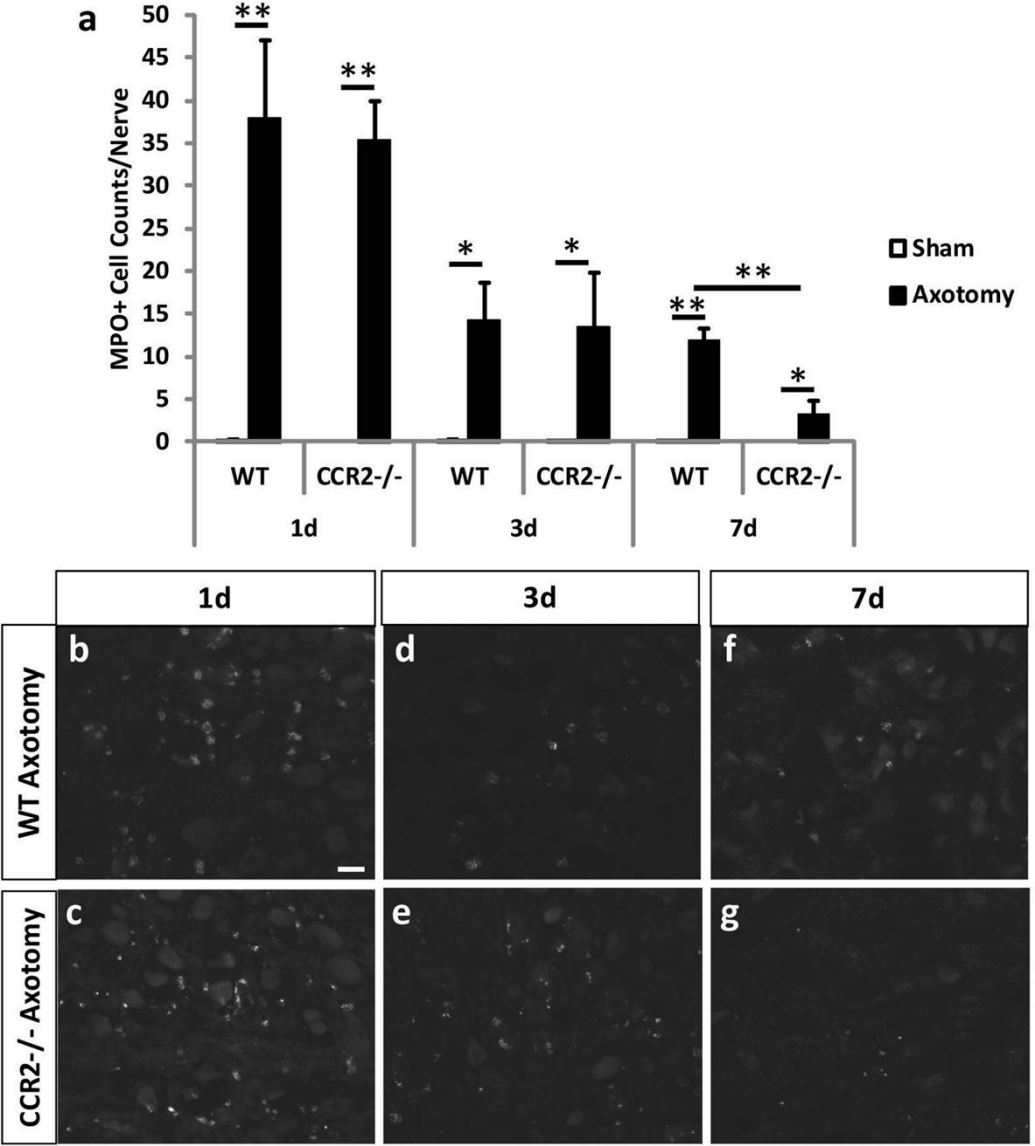
Myeloperoxidase staining of neutrophils in the axotomized SCG 1, 3, and 7 days after axotomy. Immunohistochemical staining of neutrophils with an antibody against MPO showed comparable neutrophil cell counts between WT and *Ccr2*^*−/−*^ SCG 1 and 3 days after injury and a significantly higher number of cells in WT ganglia compared to mutants 7 days after injury. Cell counts were performed using ImageJ at 1, 3, and 7 days post injury (**a**). The counts represent the sum of cells from three images per section. Representative images of IHC staining in the SCG at 1, 3, and 7 days post axotomy are shown for WT Ax (**b**, **d**, **f**) and *Ccr2*^*−/−*^ Ax (**c**, **e**, **g**). All images were taken at × 25 magnification. Scale bar = 20 μm. *n* = 5 per group. *p < 0.05. **p < 0.001

Unlike with the DRG, axotomy induced an increase in neutrophils in the SCG for both genotypes 3 days after injury (Fig. 4b, f). Interestingly, a significantly larger influx of neutrophils in the *Ccr2*^*−/−*^ SCG was observed at 3 days post injury compared to the WT SCG. To visualize the localization of neutrophils in the ganglia, immunohistochemical analyses of these polymorphonuclear cells were performed using MPO, which is a common neutrophil marker [43, 44]. While no MPO^+^ neutrophils were identified in the DRG (data not shown), significant increases in MPO^+^ neutrophil accumulation were observed in the axotomized SCG in both WT and *Ccr2*^*−/−*^ mice compared to their respective sham-operated controls (Fig. 5a–g). However, in contrast to flow cytometry data that used Ly6G and CD11b to label neutrophils (Fig. 4f), the percent of MPO^+^ neutrophils in *Ccr2*^*−/−*^ ganglia at 3 days post injury were comparable to WT ganglia (Fig. 5a, d, e). No differences in fluorescent staining were observed 1 day post injury between genotypes (Fig. 5a–c). Unexpectedly, an increase in MPO^+^ cells was observed in the WT SCG 7 days after axotomy, while expression was significantly decreased in the *Ccr2*^*−/−*^ SCG (Fig. 5a, f, g).

### Cytokine expression in the axotomized ganglia

**Fig. 6:**
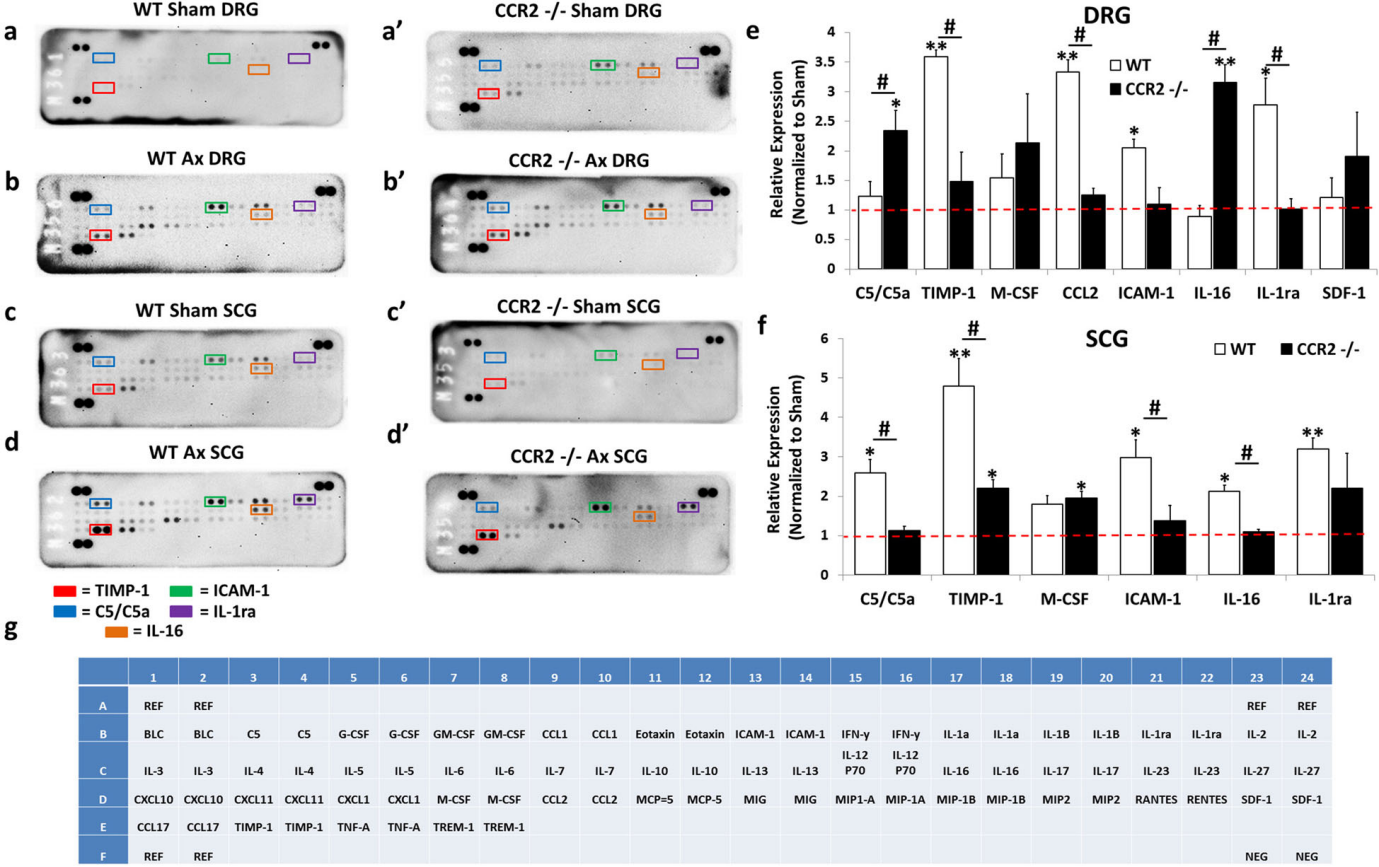
Injury-induced cytokine profiles in the DRG and SCG 7 days after axotomy. Mice underwent a unilateral transection of the right sciatic nerve and the right ICN and ECN of the SCG. Seven days after axotomy, the injured and contralateral sham-operated L4/L5 DRG and SCG were collected and flash frozen. Protein was isolated from sham and injured ganglia pooled from five mice and used for measurement of cytokines using a Proteome Profiler Mouse Array kit (**g**). Representative arrays for WT [85] and *Ccr2*^*−/−*^ (**a’**–**d’**) mice for DRG (**a**, **a’**, **b**, **b’**) and SCG (**c**, **c’**, **d**, **d’**) are shown. Array images were analyzed by densitometry for integral optical density using ImageJ. The optical density for each cytokine was then normalized to the internal control for each blot. The data are represented as the fold increase in the expression of the injured condition compared to the sham for each genotype for DRG (**e**) and SCG (**f**). The spots corresponding to the cytokines TIMP-1, sICAM-1, C5/C5a, IL-1ra, and IL-16 are highlighted in the representative arrays based on the template indicating the location of controls and various chemokine capture antibodies (**g**). *N* = 3 arrays per group. The data are presented as the mean ± SEM. *p < 0.05. **p < 0.001 represents significance of expression relative to sham within genotype. ^#^*p* < 0.05. ^##^*p* < 0.001 represents significance between genotypes

The cellular immune response in ganglia following axotomy defined by flow cytometry (Figs. 3 and 4) displayed differences in the myeloid cell population present in the DRG and SCG between WT and *Ccr2*^*−/−*^ mice 7 days after axotomy. To assess how the molecular immune response may be impacted by differences in the cellular immune populations with peripheral ganglia, the cytokine expression profile in the DRG and SCG of WT and *Ccr2*^*−/−*^ mice was assayed 7 days after axotomy. In the DRG, WT mice displayed significant injury-induced expression of multiple cytokines and immune-related factors including a 3.6-fold increase in TIMP-1, a 2.1-fold increase in sICAM-1, a 3.3-fold increase in CCL2, and a 2.8-fold increase in IL-1ra compared to WT sham DRG (Fig. 6a, b, e). In contrast, *Ccr2*^*−/−*^ mice only exhibited a significant 2.3-fold increase in C5/C5a and a 3.2-fold increase in IL-16 compared to *Ccr2*^*−/−*^ sham DRG (Fig. 6a’, b’, e). Furthermore, comparison of the DRG cytokine expression profiles between genotypes found that TIMP-1, CCL2, and IL-1ra were more highly expressed in the axotomized WT DRG compared to *Ccr2*^*−/−*^ DRG, while C5/C5a and IL-16 were more highly expressed in the *Ccr2*^*−/−*^ DRG compared to WT 7 days after injury (Fig. 6e).

In the SCG, WT mice displayed significant injury-induced expression of multiple cytokines and immune-related factors including a 2.6-fold increase in C5/C5a, a 4.8-fold increase in TIMP-1, a 3.0-fold increase in sICAM-1, a 2.1-fold increase in IL-16, and a 3.2-fold increase in IL-1ra compared to WT sham SCG (Fig. 6c, c, f). Axotomized *Ccr2*^*−/−*^ mice exhibited a significant 2.2-fold increase in the TIMP-1 and a 1.9-fold increase in M-CSF compared to *Ccr2*^*−/−*^ sham SCG (Fig. 6c’ , d’ , f). A comparison of the SCG cytokine expression profiles between genotypes found that C5/C5a, TIMP-1, IL-16, and sICAM-1 were more highly expressed in the WT SCG compared to *Ccr2*^*−/−*^ SCG 7 days after injury (Fig. 6f). The most striking differences in injury-induced expression patterns between DRG and SCG are in C5/C5a and IL-16. In the DRG, C5/C5a and IL-16 are more highly expressed in *Ccr2*^*−/−*^ mice than in WT mice; however, the expression pattern is the exact opposite in the SCG where these immune-related factors are more highly expressed by WT mice compared to *Ccr2*^*−/−*^ mice (Fig. 6e, f).

### Activation state of macrophages in peripheral ganglia following nerve injury

**Fig. 7:**
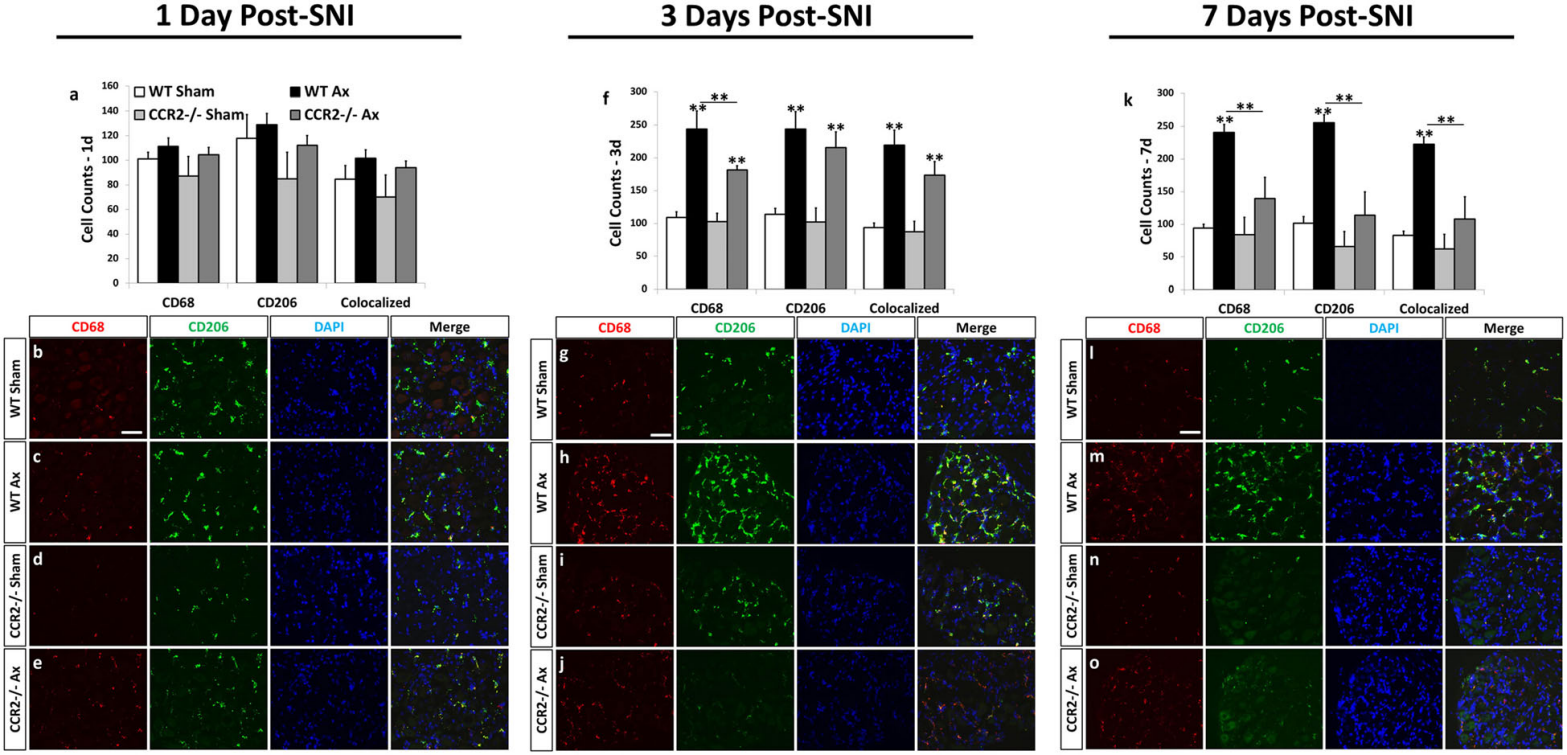
The anti-inflammatory marker, CD206, is highly expressed in CD68^+^ macrophages in the WT DRG 3 and 7 days after sciatic nerve transection. Immunohistochemical labeling of DRG sections with antibodies against a macrophage marker, CD68, and an anti-inflammatory marker, CD206, shows that a majority of macrophages in the WT DRG at 7 days post injury express CD206 (**k**). The number of CD68^+^ and CD206^+^ cells was significantly diminished in *Ccr2*^*−/−*^ mice at 3 days (**f**) and 7 days (**k**) post injury. Cell counts were performed using ImageJ at 1, 3, and 7 days post injury for CD68^+^-, CD206^+^-, and CD68^+^CD206^+^-colocalized cells (**a**, **f**, **k**). The counts represent the sum of cells from three images per section. Representative images of IHC staining in the L5 DRG at 1, 3, and 7 days post axotomy are shown for WT sham (**b**, **g**, **l**), WT Ax (**c**, **h**, **m**), *Ccr2*^*−/−*^ sham (**d**, **i**, **n**), and *Ccr2*^*−/−*^ Ax (**e**, **j**, **o**). All images were taken at × 25 magnification. Scale bar = 50 μm. *n* = 5 per group. *p < 0.05. **p < 0.001

Macrophages have been shown to increase the regenerative response of DRG neurons through releasable factors [45]; however, this ability was dependent upon the activation state of the macrophages. Conditioned medium derived from macrophages stimulated to an anti-inflammatory state was growth promoting compared to conditioned medium taken from pro-inflammatory macrophages to DRG neurons plated on inhibitory substrates [45, 46]. Given the differences in the molecular and cellular immune response between the SCG and DRG and WT and *Ccr2*^*−/−*^ mice, we sought to determine if macrophages in our system expressed an activation state which would be expected to support regeneration. Expression of the anti-inflammatory marker CD206 was assessed in the SCG and DRG at 1, 3, and 7 days after axotomy. CD206, a mannose receptor, is a useful marker of anti-inflammatory polarization as pro-inflammatory macrophages do not express this receptor [47–49]. In the DRG, significant accumulation of CD68^+^ macrophages was not seen until 3 days post injury compared to sham-operated controls (Fig. 7f–j). Macrophage accumulation was sustained at 7 days post injury, where 91.3 ± 3.7% of CD68^+^ cells colocalized with CD206 (Fig. 7k–o). *Ccr2*^*−/−*^ mice displayed a significant reduction in both CD68^+^ cells and CD68^+^CD206^+^-colocalized cells 7 days post injury (Fig. 7k). The reduction in colocalized cells in the *Ccr2*^*−/−*^ DRG does not represent a specific reduction in the expression of CD206 as 85.3 ± 5.5% of CD68^+^ cells present coexpressed CD206.

**Fig. 8:**
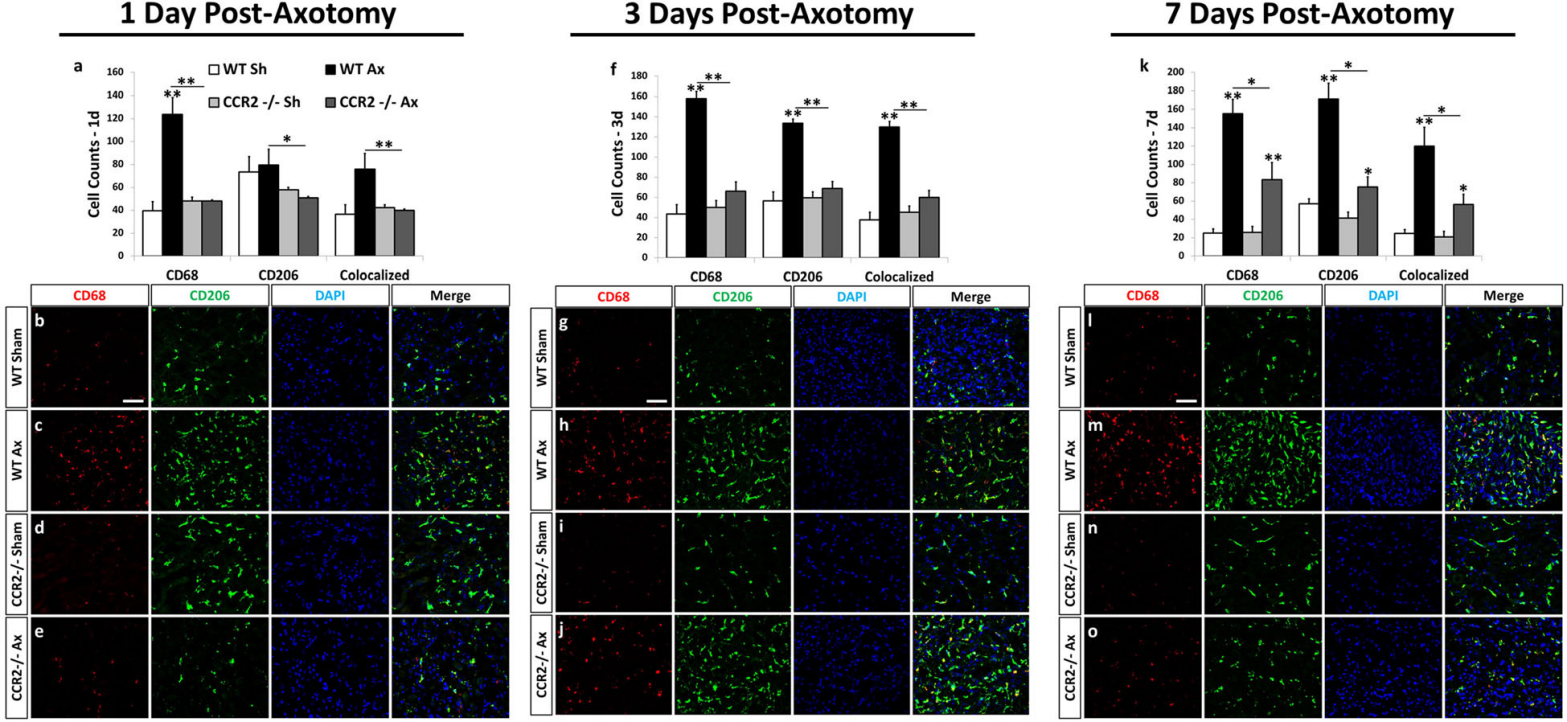
The anti-inflammatory marker, CD206, is highly expressed in CD68^+^ macrophages in the WT SCG at 1, 3, and 7 days after axotomy. Immunohistochemical labeling of SCG sections with antibodies against a macrophage marker, CD68, and an anti-inflammatory marker, CD206, shows that a majority of macrophages in the WT SCG at 3 and 7 days post injury express CD206 (**f**, **k**). The number of CD68^+^ and CD206^+^ cells was significantly diminished in *Ccr2*^*−/−*^ mice at 3 and 7 days post injury (**f**, **k**). Cell counts were performed using ImageJ at 1, 3, and 7 days post injury for CD68^+^-, CD206^+^-, and CD68^+^CD206^+^-colocalized cells (**a**, **f**, **k**). The counts represent the sum of cells from three images per section. Representative images of IHC staining in the SCG at 1, 3, and 7 days post axotomy are shown for WT sham (**b**, **g**, **l**), WT Ax (**c**, **h**, **m**), *Ccr2*^*−/−*^ sham (**d**, **i**, **n**), and *Ccr2*^*−/−*^ Ax (**e**, **j**, **o**). All images were taken at × 25 magnification. Scale bar = 50 μm. *n* = 5 per group. *p < 0.05. **p < 0.001

In the axotomized SCG, significant macrophage accumulation was detected in WT mice as early as 1 day post axotomy (Fig. 8a), an effect that was sustained out to 7 days post injury (Fig. 8k). A high level of colocalization between CD206 and CD68 is seen in that 77.6 ± 9.5% of CD68^+^ cells were also labeled with CD206. The *Ccr2*^*−/−*^ SCG displayed reduced CD68^+^ macrophage accumulation compared to WT at all time points (Fig. 8a, f, k). These data indicate that macrophages present within peripheral ganglia after nerve injury may be anti-inflammatory in nature.

## Discussion

CCL2 overexpression in sensory ganglia has been shown to be sufficient to cause macrophage accumulation in the DRG without a nerve injury [17]. Here, we have shown that CCL2 protein expression is increased in both the WT and *Ccr2*^*−/−*^ axotomized DRG and SCG early after injury. CCL2 upregulation is associated with a heightened macrophage response in both ganglia, albeit this response is markedly reduced in *Ccr2*^*−/−*^ mice. Indeed, deletion of the receptor alone showed almost complete blockade of macrophage accumulation in the DRG and a significant but only partial reduction in the SCG after nerve injury [14]. These results suggest that a second chemokine and/or action through a second chemokine receptor is facilitating monocyte-mediated chemotaxis to the injured sympathetic ganglia.

An obvious candidate for this role is MIP-1γ, which we found, using the unbiased chemokine array, to be increased only in the injured SCG of both WT and *Ccr2*^*−/−*^ mice. MIP-1γ acts through CCR1 expressed on monocytes, neutrophils, dendritic cells, and T cells [50–53]. Some evidence indicates that MIP-1γ may act in an autocrine fashion on macrophages to promote their activation and survival [53], while other studies point to dendritic cells as important producers of this chemokine for extravasation of inflammatory cells from the bloodstream [51]. Regardless of its source, heightened expression of MIP-1γ in the SCG is associated with increased macrophage accumulation. Furthermore, robust expression of both MIP-1γ and CCL2 in the SCG could explain the early and significant accumulation of macrophages that we observed in the axotomized sympathetic ganglia at 1 day post injury, compared to the typical increase in the macrophage response observed in the injured DRG after 3 days [11, 12]. However, we would need to confirm that MIP-1γ is upregulated in the SCG earlier than the 48-h time point that was used in this study.

What are the consequences of inhibiting macrophage accumulation in these ganglia after nerve injury? The most well-described function of macrophages is their role in the distal nerve segment where they promote regeneration of injured neurons through growth factor release and the removal of inhibitory axonal and myelin debris at the distal nerve that would otherwise obstruct the path for regenerating axons [54–57]. Though peripheral nerve degeneration involves the actions of multiple cells, hematogenous macrophages that infiltrate as monocytes from the blood to the injury site have been considered to be necessary for the phagocytosis of myelin and axonal fragments [13, 58–60]. It is believed that while Schwann cells initiate myelin uptake and removal [61, 62], infiltrating macrophages complete the clearance process [60, 63, 64]. However, recent studies have shown that myelin clearance is normal in *Ccr2*^*−/−*^ sciatic nerves after an injury [14], and that neutrophils play a pivotal role in this clearance [65].

Of late, a new site of macrophage action has been proposed in promoting nerve regeneration. Following injury to the sciatic nerve or ICN and ECN, macrophages accumulate within the DRG and SCG, respectively [14]. Mice lacking CCR2 exhibited diminished macrophage accumulation in the ganglia and a concomitant decrease in axonal regeneration [14], while a similar study that administered the antibiotic minocycline to reduce macrophage numbers in the DRG also observed a reduction in regeneration [11]. Moreover, our lab provided evidence that simply overexpressing CCL2 in sensory ganglia without a nerve injury is sufficient to mount an injury-like macrophage response in the DRG and to prompt robust neurite growth [17].

What remains to be determined is how these macrophages promote regeneration at the level of the neuronal cell bodies. The most likely mechanism of action is through a macrophage-derived releasable factor, such as a cytokine, that stimulates the neuronal expression of regeneration-associated genes [45, 66–68]. Analysis of regeneration-associated genes in the uninjured DRG in which CCL2 was overexpressed highlighted increased expression of leukemia inhibitory factor (*Lif*) mRNA [17], a gene that had been previously described as promoting sensory and sympathetic neuron regeneration [69, 70].

In the cytokine array, we observe differential expression of cytokines between genotypes in the injured DRG 7 days after injury, many of which are downregulated in the *Ccr2*^*−/−*^ model compared to WT mice. This is not surprising if we consider evidence that the lack of CCR2 and a resulting decrease in macrophage accumulation in the injured ganglia reveal an inhibition of in vitro regeneration of both sensory and sympathetic neurons [14]. TIMP-1, a protein that we find is reduced in the axotomized *Ccr2*^*−/−*^ DRG and SCG compared to their axotomized WT counterparts, has been shown to be important in supporting regeneration in the central nervous system [71]. TIMP-1 functions to inhibit the activity of metalloproteinases which cleave myelin-associated glycoproteins that accumulate in the central nervous system after nerve injury and obstruct the path of regenerating DRG neurons [72]. Peripheral nerve injury has been shown to induce TIMP-1 expression in the DRG [73]. Furthermore, a preconditioning injury to the peripheral nerve process of the DRG followed by an injury to the central nerve process results in TIMP-1 upregulation in the DRG and the spinal cord in addition to increased neurite outgrowth into the spinal cord [71].

The specific contribution of macrophage releasable factors to the cytokine microenvironment has been shown to be indicative of the macrophage phenotype, i.e., pro-inflammatory or anti-inflammatory [46]. Pro-inflammatory macrophages are characterized by the increased production of nitric oxide and specifically the enzyme inducible nitric oxide synthase (iNOS) [74, 75], while anti-inflammatory macrophages upregulate expression of the mannose receptor CD206 [76]. These distinctions of macrophage activation states are important because of recent work that has shown that conditioned media taken from anti-inflammatory macrophage cultures was able to stimulate DRG neurite outgrowth on both growth-permissive and growth-inhibiting substrates [45]. We previously reported increased CD206 transcript levels in the uninjured DRG of WT mice where CCL2 was overexpressed, resulting in increased macrophage accumulation and neurite outgrowth [17]. Here, we find that a majority of macrophages in the axotomized WT DRG and SCG at 1, 3, and 7 days post injury express the anti-inflammatory marker CD206, which suggests that these macrophages are of an anti-inflammatory nature. It should be noted that most macrophages in the axotomized *Ccr2*^*−/−*^ DRG and SCG also express CD206, but the overall accumulation of these myeloid cells is drastically reduced relative to WT mice. Furthermore, the hypothesis that monocyte-derived macrophages that infiltrate the DRG and SCG after injury are of an anti-inflammatory type is evidence of the small number of macrophages that express the pro-inflammatory marker iNOS (data not shown).

In addition to the evaluation of macrophage activation states and the evident differences in macrophage accumulation between genotypes in the axotomized ganglia, we also note a genotype-specific alteration in neutrophil chemotaxis to the SCG. *Ccr2*^*−/−*^ sympathetic ganglia boast a larger neutrophil population than its WT counterpart 3 days following nerve injury. This increase is associated with heightened expression of CXCL5, a neutrophil chemokine, in the *Ccr2*^*−/−*^ SCG [77]. Interestingly, upregulation of another neutrophil chemokine MIP-2 (CXCL2) was not observed, though this chemokine most likely plays a role in increased neutrophil accumulation in the injured *Ccr2*^*−/−*^ sciatic nerve compared to WT nerves [65].

Interestingly, our data suggest that different subpopulations of neutrophils may exist in the SCG after nerve injury. Flow cytometry analysis demonstrated that CD11b^+^Ly6G^+^ neutrophils were significantly more abundant in the *Ccr2*^*−/−*^ SCG 3 days following injury compared to the WT SCG; however, IHC data showed comparable MPO^+^ neutrophil accumulation in the sympathetic ganglia between genotypes at this same time point. The idea of neutrophil heterogeneity has gained considerable traction with evidence that has shown that these polymorphonuclear cells develop distinct phenotypes in response to a range of stimuli [78].

Unlike in the SCG, we do not identify a significant population of neutrophils in the DRG after sciatic nerve transection in either genotype. However, the role of neutrophils in the DRG has been extensively characterized in experimental paradigms of neuropathic pain. Neutrophils reportedly infiltrate both the DRG and the sciatic nerve in response to chronic nerve constriction [79] and partial nerve ligation [2]. These types of injuries manifest neuropathic pain due to the sensitization of neurons by exposure to a prolonged inflammatory response [1]. Neutrophils and other immune cells including macrophages and T cells have been shown to contribute to neuropathic pain via the release of TNF-α, IL-1β, and IL-6 through directly engaging cognate receptors on sensory neurons (for a summary, see [80]). Chemotactic disruption of these immune cells using pharmacological or genetic methods has been shown to ameliorate the severity of neuropathic pain [81–83].

Clear differences in chemokine and cytokine expression profiles were observed between DRG and SCG following axotomy. Forty-eight hours after injury, C5/C5a is significantly increased in the WT DRG but is not upregulated in the WT SCG, while MIP-1γ is highly upregulated in the injured WT SCG but is not increased in the DRG after injury. Seven days after injury, IL-1ra is significantly upregulated in the WT SCG but not the WT DRG. The distinct expression of specific immune signaling molecules between ganglia could be a result of the presence of a significant neutrophil population found in the SCG but not the DRG 3 days after injury. However, other ganglion-specific aspects such as the presence of a blood-ganglion barrier in the SCG or the significant heterogeneity of neurons in the DRG could play a contributing factor [24, 84].

## Conclusions

Through the characterization of cellular components that populate sensory and sympathetic ganglia in WT and *Ccr2*^*−/−*^ mice, which, in turn, influence changes in the cytokine microenvironment surrounding the axotomized neurons, we can begin to carve out unexplored territories for future research. Here, we have identified differences in the macrophage response between genotypes after injury in both DRG and SCG, but there remains a similarity of macrophage phenotype (i.e., anti-inflammatory). Additionally, a probable alternative compensatory chemokine network (MIP-1γ) that may facilitate monocyte extravasation to the injured SCG in the absence of CCR2 expression has emerged from this study. CCR2 deletion also induced increased neutrophil accumulation in the injured SCG, but the consequence of this increase is as yet unknown. Taken together, this study has revealed important molecular and cellular differences between WT and *Ccr2*^*−/−*^ mice and between axotomized DRG and SCG and has posed interesting new questions for future studies.

## Abbreviations

ANOVA: Analysis of variance
ATF3: Activating transcription factor 3
CCL2: C-C class chemokine ligand 2
CCR2: C-C class chemokine receptor 2
DRG: Dorsal root ganglion
ECN: External carotid nerve
ICN: Internal carotid nerve
IHC: Immunohistochemistry
IL: Interleukin
IL-1ra: Interleukin-1 receptor antagonist
iNOS: Inducible nitric oxide synthase
LIF: Leukemia inhibitory factor
MCP-1: Monocyte chemoattractant protein-1
MIP-1γ: Macrophage inflammatory protein-1 gamma
MPO: Myeloperoxidase
PNS: Peripheral nervous system
SCG: Superior cervical ganglion
sICAM-1: Soluble intercellular adhesion molecule-1
TIMP-1: Tissue inhibitor of metalloproteinase-1
TNF-α: Tumor necrosis factor alpha
WT: Wild type
